# Anatomical variation of inner ear may be a predisposing factor for unilateral Ménière’s disease rather than for ipsilateral delayed endolymphatic hydrops

**DOI:** 10.1007/s00330-021-08430-7

**Published:** 2022-01-03

**Authors:** Ping Lei, Yangming Leng, Jing Li, Renhong Zhou, Bo Liu

**Affiliations:** 1 Department of Radiology, Union Hospital, Tongji Medical College, Huazhong University of Science and Technology, Wuhan, 430022 China; 2 Department of Otorhinolaryngology, Union Hospital, Tongji Medical College, Huazhong University of Science and Technology, Wuhan, 430022 China

**Keywords:** Ménière’s disease, Endolymphatic hydrops, Magnetic resonance imaging, Endolymphatic sac

## Abstract

**Objective:**

Radiological anatomical variations, measured by magnetic resonance imaging (MRI), were evaluated in patients with ipsilateral delayed endolymphatic hydrops (DEH) and unilateral Ménière’s disease (MD). The role of anatomical variations in different subtypes of hydropic ear disease was investigated.

**Methods:**

Twenty-eight patients with ipsilateral DEH, 76 patients with unilateral MD, and 59 control subjects were enrolled. The radiological indices included the distance between the vertical part of the posterior semicircular canal and the posterior fossa (MRI-PP distance) and the visibility of vestibular aqueduct (MRI-VA). These variations among patients with DEH, MD, and control subjects were compared. The correlation between radiological anatomical variations and clinical features or audio-vestibular findings was also examined.

**Results:**

(1) MRI-PP distance in the affected side of unilateral MD was shorter than that in ipsilateral DEH (*Z* =  − 2.481, *p* = 0.013) and control subjects (*Z* =  − 2.983, *p* = 0.003), while the difference of MRI-PP distance between the affected side of ipsilateral DEH and control subjects was not statistically significant (*Z* =  − 0.859, *p* = 0.391). (2) There was no significant interaural difference of MRI-PP distance in patients with unilateral MD (*Z* =  − 0.041, *p* = 0.968) and ipsilateral DEH (*t* =  − 0.107, *p* = 0.915) respectively. (3) No significant interaural difference of MRI-VA visibility was observed in patients with unilateral MD (χ^2^ = 0.742, *p* = 0.389) and ipsilateral DEH (χ^2^ = 0.327, *p* = 0.567) respectively. (4) No correlation was found between these anatomical variables and clinical features or audio-vestibular findings in patients with unilateral MD and ipsilateral DEH respectively (*p* > 0.05).

**Conclusions:**

Anatomical variations of inner ear may be a predisposing factor in the pathogenesis of unilateral MD rather than ipsilateral DEH.

**Key Points:**

*• Patients with ipsilateral delayed endolymphatic hydrops showed normal distance between the vertical part of the posterior semicircular canal and the posterior fossa.*

*• Compared to patients with ipsilateral delayed endolymphatic hydrops and control subjects, patients with unilateral Ménière’s disease exhibited shorter distance between the vertical part of the posterior semicircular canal and the posterior fossa.*

*• Anatomical variations of inner ear may be a predisposing factor in the pathogenesis of unilateral Ménière’s disease rather than ipsilateral delayed endolymphatic hydrops.*

**Supplementary Information:**

The online version contains supplementary material available at 10.1007/s00330-021-08430-7.

## Introduction

Ménière’s disease (MD) is a relatively common and debilitating otological condition characterized by repetitive vertiginous episodes, fluctuant sensorineural hearing loss (SNHL), tinnitus, and aural fullness. The pathological hallmark of MD is endolymphatic hydrops (ELH), although its role in the pathophysiology of the disease remains unclear. Many factors have been proposed as leading to the development of ELH, which involve excessive endolymph production and decreased endolymph absorption by the endolymphatic sac (ES), ionic imbalance, genetic predisposition, anatomical abnormalities, viral infection, autoimmune reactions, vascular irregularities, allergic responses, and others [1]. Among them, the anatomical variations of the inner ear have been studied histopathologically and radiologically [2]. ES and vestibular aqueduct (VA) have been demonstrated to be significantly smaller in MD patients than in healthy individuals by the temporal bone histopathological surveys [3, 4]. Furthermore, radiological studies have found various anatomical variations of the inner ear in MD patients, as visualized on magnetic resonance imaging (MRI) or computed tomography (CT), including reduced distance between the vertical part of the posterior semicircular canal and the posterior fossa, less visibility of endolymphatic duct (ED) or VA [5–7], poor periaqueductal pneumatization [8], higher prevalence of jugular bulb abnormalities [9], retro-vestibular bony hypoplasia [10], and so forth.

Delayed endolymphatic hydrops (DEH), another subtype of hydropic ear disease [11], is a rare clinical entity characterized by episodic vertigo of delayed onset following profound SNHL, whose otological symptoms are similar to those of MD [12]. Clinically, DEH can be categorized into ipsilateral and contralateral types. Ipsilateral type refers to profound SNHL followed by episodic vertigo. On the other hand, contralateral type exhibits similar features to ipsilateral DEH as well as fluctuating hearing loss in the opposite better-hearing ear, sometimes with episodic vertigo attacks [13]. Histopathologically, both DEH and MD shared the characteristics of ELH [14]. The cause of DEH is unknown, and the development is assumed to be bi-phasic: (1) a labyrinthine insult of sufficient magnitude to damage the cochlear totally while preserving the vestibular function and (2) delayed atrophy or fibrous obliteration of the endolymphatic resorptive system of the membranous labyrinth [12, 15]. To date, the research on the relationship between anatomical variations and hydropic ear disease mainly focuses on MD, the idiopathic ELH [2, 5]. What role does the anatomical variations of the inner ear play in the development of DEH? This question remains elusive until now.

In this study, the MRI evaluations were respectively reviewed in patients with ipsilateral DEH and unilateral MD. We aimed to examine the differences in MRI-visualized anatomical variations of the inner ear between these two disorders. Also, the relationships between the radiological findings and clinical features or audio-vestibular results were explored.

## Materials and methods

### Participants

A retrospective chart review was conducted in the Union Hospital, Tongji Medical College, Huazhong University of Science and Technology, Wuhan, China.

Twenty-eight patients with ipsilateral DEH and 76 patients with unilateral definite MD were enrolled between September 2012 and December 2019. For all patients, a thorough history inquiry, otoscopy, neurotological evaluations (audiometry, impedance, videonystagmograph, caloric test, etc.), and imaging examination were conducted for differential diagnosis. The diagnosis of MD was established following the diagnostic guidelines of MD outlined by the American Academy of Otolaryngology-Head and Neck Surgery (AAO-HNS) in 1995 [16]. The ipsilateral DEH was diagnosed against the criteria formulated by the committee of the Japan Society for Equilibrium Research in 1987 [17]: (1) a precedent profound SNHL in one ear; (2) delayed development of vertigo attacks without fluctuating hearing loss in the opposite ear; and (3) exclusion of central nervous system lesions, eighth nerve tumors, and other cochleovestibular diseases such as syphilitic labyrinthitis. Profound SNHL was defined as a pure tone average of greater than 90 dB over the 0.5-kHz, 1.0-kHz, and 2.0-kHz frequencies. Fifty-nine subjects without audio-vestibular symptoms were enrolled as the control group.

The exclusion criteria were as follows: (1) middle or inner ear infections (otitis media, mastoiditis, labyrinthitis, etc.); (2) middle or inner ear anomaly; (3) having received previous ear surgery or intratympanic injections; (4) retro-cochlear lesions (vestibular schwannoma, internal acoustic canal stenosis, etc.); (5) bilateral MD; (6) head trauma; (7) systemic diseases; (8) disorders of central nervous system (vestibular migraine, multiple sclerosis, cerebellar infarction, etc.).

This study was conducted in accordance with the tenets of the Declaration of Helsinki. The study was approved by the Ethical Committee of Union Hospital, Tongji Medical College, Huazhong University of Science and Technology, Wuhan, China.

## Methods

### Audio-vestibular evaluations

For all patients included, the pure tone audiogram was performed during the interictal period. Furthermore, some patients received additional audio-vestibular evaluations, including the electrocochleogram (EcochG), caloric test, and audiometric glycerol test. All subjects were instructed not to take alcohol, caffeine, or medications (sedative, anti-depressant drugs, etc.) that could affect the results of vestibular tests within 48 h before vestibular testing.

The stage of MD was determined according to the AAO-HNS guidelines (1995) [16]. Comprehensive audio-vestibular evaluations, including EcochG, audiometric glycerol test, and caloric test, were performed as described by previous literature [18]. For the EcochG, summating potential (SP) and action potential (AP) were recorded, and the SP/AP ratio was calculated. The SP/AP ratio ≥ 0.4 was deemed as positive. The results of audiometric glycerol test were deemed positive when the pure-tone hearing threshold was decreased by (1) at least 10 dB at any three or more frequencies or (2) at least 15 dB at one frequency at any time point after glycerol intake. During the caloric test, the maximum slow phase velocity (SPV_max_) of caloric nystagmus was measured following each air irrigation, and the canal paresis (CP) was calculated following Jongkees’ formula. If the interaural asymmetry of the caloric nystagmus was ≥ 25%, the result was considered significant, indicating abnormal caloric response. According to the published criteria [19], if the summated SPV_max_ of the induced nystagmus was < 20°/s after 4 air irrigations, the caloric response is believed to indicate bilateral vestibular hypofunction. In this case, ice water irrigation (4 °C, 1.0 ml) would be used to confirm the caloric unresponsiveness.

### Radiological evaluations

All participants received MRI examination by the Verio or Magnetom Trio 3 T scanners (Siemens) with a 12-element phased array coil. T1-weighted and T2-weighted spin-echo imaging was used. Three-dimensional sampling perfection with application optimized contrasts using different flip angle evolutions (3D-SPACE) was used to quantify the distance between the vertical part of the posterior semicircular canal and the posterior fossa (Supplementary Table 1 in supplementary materials).

**Fig. 1:**
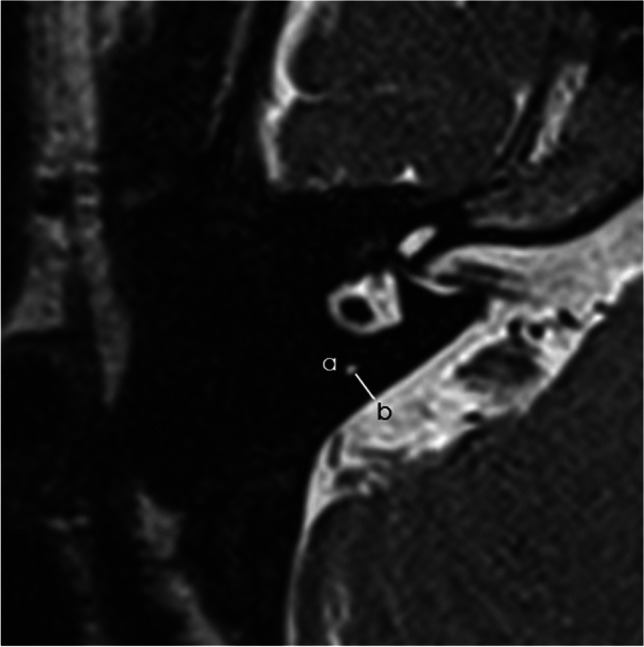
A 0.5-mm axial 3D-SPACE MRI scan showing detailed image of the right ear at the level of the measured distance between the vertical part of the posterior semicircular canal (a) and the posterior fossa (b)

**Fig. 2:**
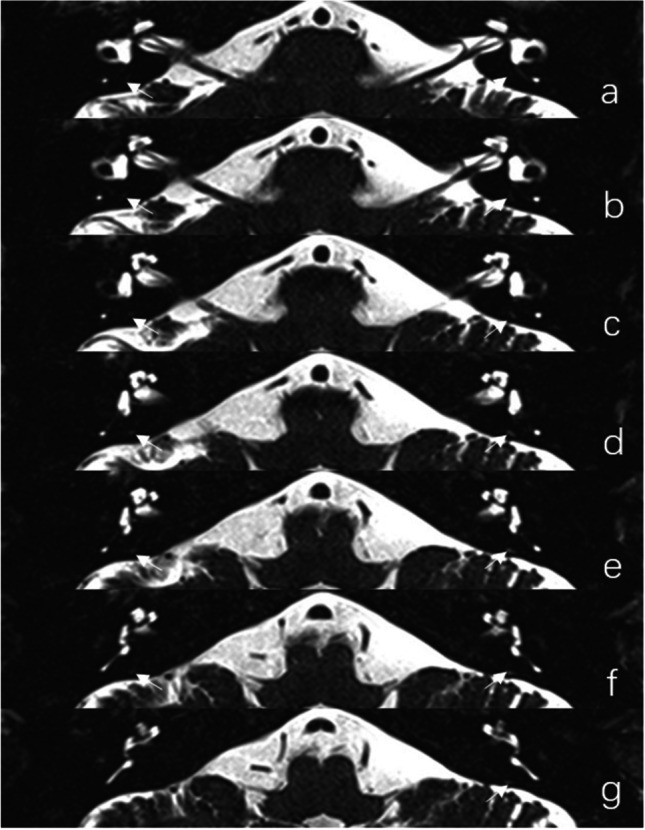
3D-SPACE MRI images of a 63-year-old male in control group. (**a**, **b**, **c**, **d**, **e**, **f**, **g**) Axial, high-resolution, T2-weighted MRI scan showing visualization of the vestibular aqueduct on both sides

**Fig. 3:**
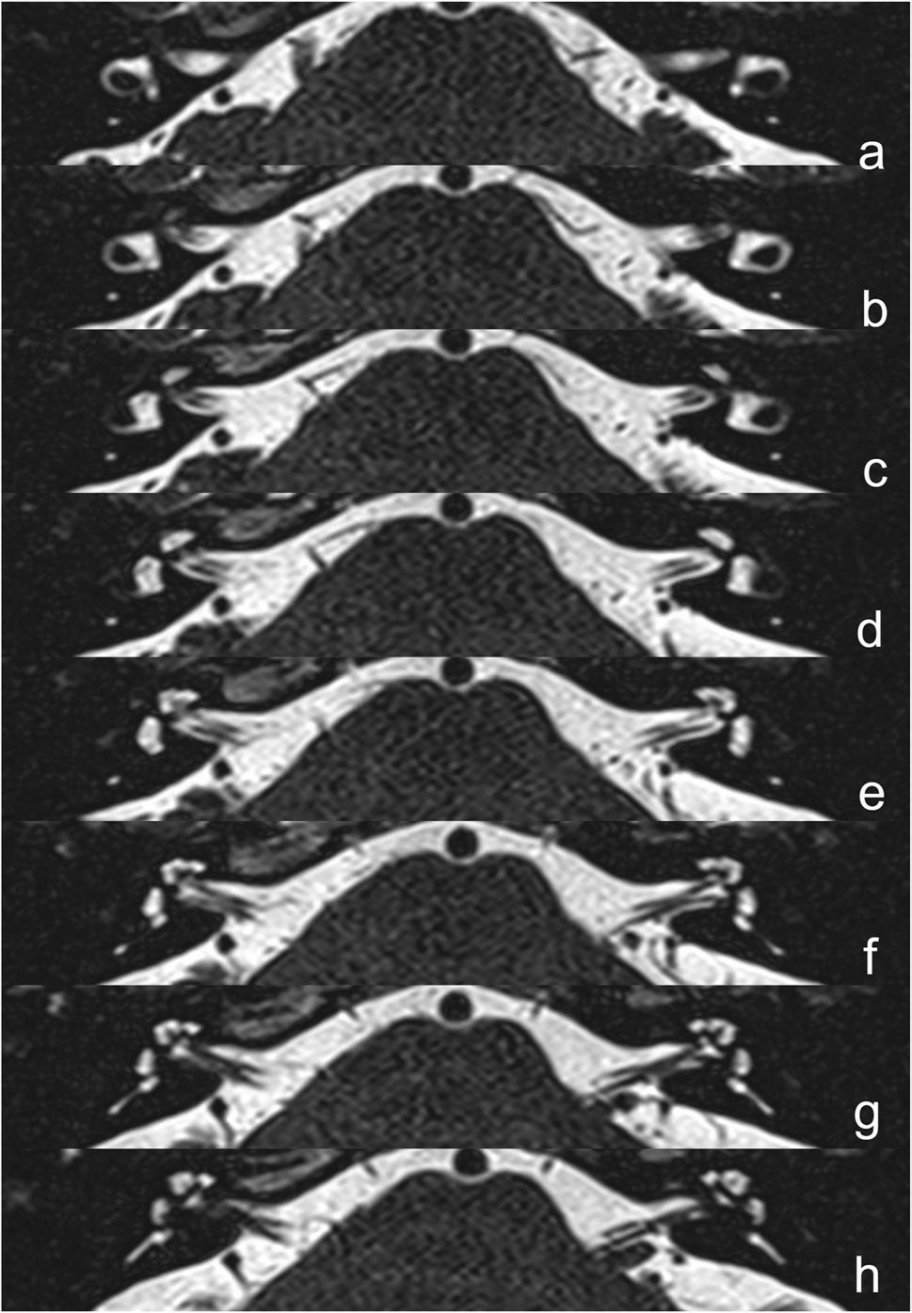
3D-SPACE MRI images of a 53-year-old male with left-sided unilateral MD. (**a**, **b**, **c**, **d**, **e**, **f**, **g**, **h**) Axial, high-resolution, T2-weighted MRI scan showing non-visualization of the vestibular aqueduct on both sides

All MRI data were transferred to the workstations and imaging analyses were performed on a picture archiving and communication system (PACS) workstation (Carestream Client, Carestream Health). Radiological data of patients and control subjects were intermixed and reviewed by two senior neuroradiologist who were blinded to the clinical data (L.P. with an experience of over 10 years and L.J. of over 5 years). In this study, anatomical variations by MRI-visualized measurement included the distance between the vertical part of the posterior semicircular canal and the posterior fossa (MRI-PP distance) and visualization of VA (MRI-VA visibility). MRI-PP distance was evaluated at 3D-SPACE axial images, which were parallel to cochleovestibular nerve of both sides in coronal position and the positioning line is inclined about 15 degrees forward and downward in sagittal position. The shortest distance between the vertical part of the posterior semicircular canal and the posterior fossa was measured (presented in Fig. 1). Visibility of VA refers to that a linear or dot-like high intensity is visualized continuously on more than one MRI sections in the direction of common crus to the posterior edge of the temporal bone. Typical examples of visualization and non-visualization of VA in 3D-SPACE are presented in Fig. 2 and Fig. 3 respectively.

### Statistical analyses

Statistical analyses were performed by using software SPSS (version 22.0) and drawing is done by software R (version 3.5.2). All continuous variables are presented as means ± standard deviations (SDs) or median and interquartile range (IQR 25th to 75th percentiles). Categorical variables are presented as counts and percentages. The interobserver agreement for MRI-measurement variables was determined using the intraclass correlation efficient (ICC). The level of agreement was generally recognized as follows: poor, ICC < 0.20; fair, 0.2 < ICC ≤ 0.40; moderate, 0.4 < ICC ≤ 0.60; good, 0.6 < ICC ≤ 0.80; and excellent, 0.8 < ICC ≤ 1.0.

If distribution of MRI-PP distance was normal, the *t* test was used to compare between affected and non-affected side in patients. Otherwise, the Wilcoxon rank test was conducted. The chi-square test was used to compare the visualization of VA between affected and non-affected side in MD or DEH patients. The MRI-PP distance among the MD, DEH, and control subjects was compared by Kruskal–Wallis test. And the Wilcoxon rank test was used to compare the MRI-PP distance between the patients with MD or DEH and control subjects respectively. The MRI-VA visibility among the MD, DEH, and control subjects was also analyzed by chi-square test. The significance level was set at 0.05.

Multiple linear regression was used to evaluate the possible relationships between the MRI-PP distance and patients’ age, gender, course duration, Ménière’s stage, and CP value in patients with unilateral MD. Using logistic regression, the relationships between the visibility of MRI-VA and clinical features or audio-vestibular findings were investigated. For the ipsilateral DEH, these two regression analyses were also used to evaluate the relationship between the anatomical variations and patients’ age, gender, course duration, and CP value. Because there were many missing items in the results of EcochG and glycerol tests in some MD patients, the correlations between these anatomical variations and results of EcochG and glycerol tests were analyzed by *t* test or chi-square test.

## Results

### Demographic data of the participants

The demographic data of the patients with unilateral MD and ipsilateral DEH are presented in Table 1. In ipsilateral DEH group, 28 patients were enrolled, of which 18 (64.3%) were female and 10 (35.7%) were male. The median age was 32 (23.5 ~ 46.25) years old. The median disease duration was 1 (0.5 ~ 3) year. In the unilateral MD group, 76 patients were enrolled, of which 44 (57.9%) were female and 32 (42.1%) were male. The median age was 49 (42 ~ 57.5) years old. The median disease duration was 2 (0.85 ~ 5) years. Among the MD patients, 3 cases were classified as Ménière’s stage I, 20 cases stage II, 45 cases stage III, and 8 cases stage IV (Table 1).

**Table 1:** Demographic features of the patients with unilateral MD and ipsilateral DEH

	Unilateral MD	Ipsilateral DEH
Age (years)	49 (42 ~ 57.5)	32 (23.5 ~ 46.25)
Gender (female/male)	44/32	18/10
Course duration (years)	2 (0.85 ~ 5)	1 (0.5 ~ 3)
Affected ear (left/right)	44/32	9/19
Stage of MD (I/II/III/IV)	3/20/45/8	NA

*MD* Ménière’s disease, *DEH* delayed endolymphatic hydrops, *NA* not applicable

Among the ipsilateral DEH patients, twenty-four cases underwent the caloric test, and abnormal results were found in 17 cases (70.8%). Because of the profound SNHL in the affected ear, the EcochG and audiometric glycerol test was not performed. Among the unilateral MD patients, sixty-six cases underwent caloric test, and abnormal results were detected in 33 cases (50%). Sixty-one patients underwent EcochG, and the wave forms with clear SP and AP peaks were obtained in 53 patients, in which 38 cases yielded positive and 15 negative results. Furthermore, 38 cases received the audiometric glycerol test, in which 25 had positive and 13 negative results.

### Radiological evaluations

In this study, the interobserver agreement for radiological assessment was excellent for MRI-PP distance (ICC = 0.981) and MRI-VA visibility (ICC = 0.831) respectively (Supplementary Table 2 in supplementary materials), and the results evaluated by one neuroradiologist (L.P.) were used for further analyses.

#### Radiological variations in patients with MD and DEH

**Fig. 4:**
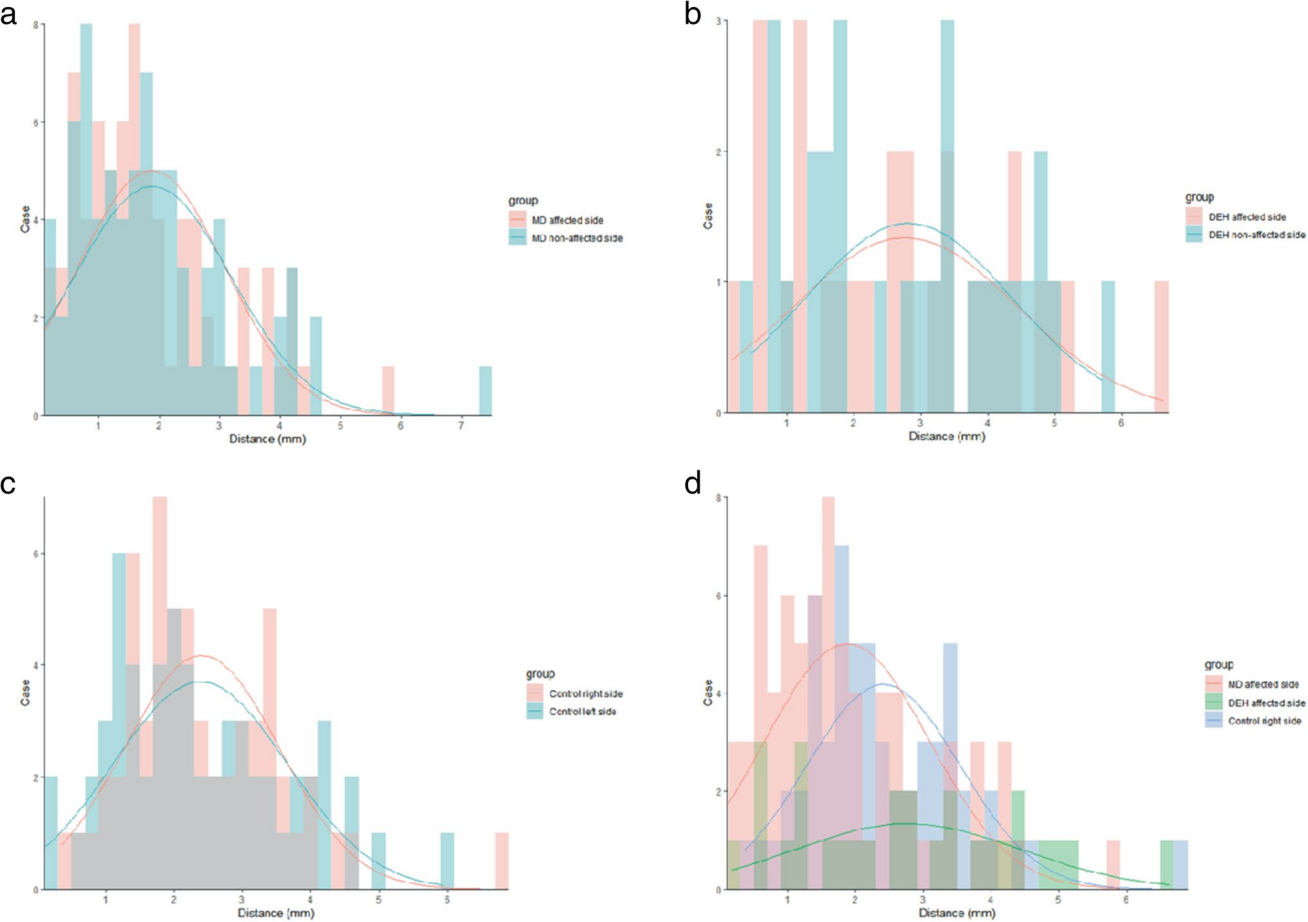
Bar graph showing the distribution of 3D-SPACE MRI-PP distances in patients with unilateral MD (**a**, including affected and non-affected side), ipsilateral DEH (**b**, including affected and non-affected side), control subjects (**c**, including right and left side), and comparison among patients with MD, DEH, and control group (**d**, including the affected side of MD, DEH, and the right side of control subjects). The curve is fitted according to the histogram and reflects the distribution of variables

Of 76 patients with unilateral MD, the mean MRI-PP distance in the affected and non-affected ears was 1.871 ± 1.211 mm and 1.891 ± 1.296 mm respectively, which were not significantly different (*Z* =  − 0.041, *p* = 0.968) (shown in Fig. 4a). The percentage of MRI-VA visibility in the affected side was 14.5% (11/76) and 19.7% (15/76) in the non-affected side, which were not significantly different (χ^2^ = 0.742, *p* = 0.389).

Of 28 patients with ipsilateral DEH, the affected ears had a mean MRI-PP distance of 2.615 ± 1.659 mm, while non-affected side had 2.578 ± 1.345 mm. No significant difference of MRI-PP distance was found between the affected and non-affected side (*t* =  − 0.107, *p* = 0.915) (shown in Fig. 4b). The percentage of MRI-VA visibility in the affected side was 35.7% (10/28) and 25.0% (7/28) in the non-affected side, which were not significantly different (χ^2^ = 0.327, *p* = 0.567).

#### Comparison of radiological variations among patients with hydropic ear diseases and control subjects

Since there was no significant interaural difference in terms of MRI-PP distance (right ear: 2.407 ± 1.127 mm, left ear: 2.382 ± 1.872 mm, *t* = 0.217, *p* = 0.829) and MRI-VA visibility (right ear: 30.5%, left ear: 28.8%, χ^2^ = 0.041, *p* = 0.840) in control subjects, data from the right ear of control subjects were selected randomly for statistical analysis (shown in Fig. 4c and Table 2). The MRI-PP distance and MRI-VA visibility in the affected ears of MD patients, DEH patients, and the right ear of control subjects were compared, and the statistical differences among the three groups are shown (in Table 2 and Fig. 4d). The results of pairwise comparisons between each two of these three groups were as follows, and group comparisons were performed with a Bonferroni’s correction using an alpha level of 0.05/3 = 0.0167. (1) The MRI-PP distance of the affected side of unilateral MD was statistically shorter than that of ipsilateral DEH (*Z* =  − 2.481, *p* = 0.013). The MRI-VA visibility of the affected side between the unilateral MD and ipsilateral DEH was not significantly different (χ^2^ = 5.729, *p* = 0.017). (2) The MRI-PP distance of the affected side of unilateral MD patients was significantly shorter than that of the right side of control subjects (*Z* =  − 2.983, *p* = 0.003). The MRI-VA visibility between the affected side of MD and the right ear of control subjects was not significantly different (χ^2^ = 4.155, *p* = 0.042). (3) No significant differences were found between the affected side of DEH and the right ear of control subjects in terms of the MRI-PP distance (*Z* =  − 0.859, *p* = 0.391) and MRI-VA visibility (χ^2^ = 0.422, *p* = 0.516), respectively.

**Table 2:** Comparison of MRI-PP distance and MRI-VA visibility among the patients with unilateral MD, ipsilateral DEH and control subjects

	MD affected side	DEH affected side	Control (right)	*H*/χ^2^	*p*
MRI-PP distance, mm	1.871 ± 1.211	2.767 ± 1.670	2.407 ± 1.127	11.586	0.003*
MRI-VA visibility, cases (%)	11 (14.5%)	10 (35.7%)	17 (28.8%)	6.729	0.035*

*MD* Ménière’s disease, *DEH* delayed endolymphatic hydrops; *MRI-PP Distance* distance between the vertical part of the posterior semicircular canal and the posterior fossa visualized by MRI, *MRI-VA visibility* visualization of the vestibular aqueduct by MRI

^*^Significant difference

### Radiological variations and the clinical profiles

For patients with unilateral MD, regression analyses revealed no significant relationship between these MRI-visualized anatomical variations (the MRI-PP distance and VA visibility) and patients’ age, gender, course duration, stage of MD, and CP value (Table 3). Furthermore, by chi-square test and *t* test, no relationships were found between the MRI-visualized anatomical variations and results of EcochG and audiometric glycerol test (Table 4). For patients with ipsilateral DEH, multiple linear regression analyses revealed no significant relationship between the MRI-PP distance and patients’ age, gender, course duration, and CP value, as there was no statistical significance in the whole regression equation model. Using the logistic regression, no relationship was demonstrated between the MRI-VA visibility and age, gender, course duration, and CP value (Table 5).

**Table 3:** Results of regression analyses between MRI-visualized anatomical variations, and patients’ age, gender, course duration, Ménière’s stage, and CP value in patients with unilateral MD

	MRI-PP distance*			MRI-VA visibility^#^		
	β	T/F	*p*	β	Wald/χ^2^	*p*
Age	− 0.001	− 1.019	0.906	0.017	0.410	0.522
Gender	− 0.299	− 0.119	0.312	− 0.392	0.313	0.576
Course	0.007	0.239	0.812	− 0.026	0.151	0.697
Stage	− 0.369	− 1.456	0.150	0.000	0.000	1.000
CP value	0.005	0.867	0.389	− 0.006	0.249	0.618
Constant	3.265	3.374	0.001	1.864	0.669	0.413
Model		0.777	0.569		1.040	0.959

*MD* Ménière’s disease, *CP* canal paresis, *MRI-PP Distance* distance between the vertical part of the posterior semicircular canal and the posterior fossa visualized by MRI, *MRI-VA visibility* visualization of the vestibular aqueduct by MRI

^*^By multiple linear regression

^#^By logistic regression

**Table 4:** Relationships between MRI-visualized anatomical variations, and results of EcochG and glycerol test in patients with unilateral MD

Results	MRI-PP distance		MRI-VA visibility	
	*t*	*p*	χ^2^	*p*
EcochG	− 0.848^a^	0.400	0.781^b^	0.377
Glycerol test	0.237^a^	0.814	0.495^b^	0.482

*MD* Ménière’s disease, *EcochG* electrocochleogram, *MRI-PP Distance* distance between the vertical part of the posterior semicircular canal and the posterior fossa visualized by MRI, *MRI-VA visibility* visualization of the vestibular aqueduct by MRI

^a^By independent-sample *t* test

^b^By chi-square test

**Table 5:** Results of regression analyses between MRI-visualized anatomical variations, and patients’ age, gender, course duration, and CP value in patients with ipsilateral DEH

	MRI-PP distance*			MRI-VA visibility^#^		
	β	T/F	*p*	β	Wald/χ^2^	*p*
Age	− 0.056	− 2.366	0.027	0.052	1.965	0.161
Gender	0.957	1.402	0.174	0.517	0.280	0.597
Course	0.028	0.389	0.701	0.023	0.029	0.865
CP value	0.000	0.025	0.980	− 0.021	1.431	0.232
Constant	3.134	2.428	0.023	− 1.130	0.367	0.545
Model		2.000	0.128		4.329	0.363

*DEH* delayed endolymphatic hydrops, *CP* canal paresis, *MRI-PP Distance* distance between the vertical part of the posterior semicircular canal and the posterior fossa visualized by MRI, *MRI-VA visibility* visualization of the vestibular aqueduct by MRI

^*^By multiple linear regression

^#^By logistic regression

## Discussion

### Radiological differences between MD and DEH

Our study found that the MRI-PP distance, which has been accepted as a measure of the size of the ES [5], was reduced in ears with unilateral MD compared with those with ipsilateral DEH and control subjects. Anatomic variations of VA and ES already exist in the newborn and develop fully in early childhood [20]. Therefore, MRI-PP distance may reflect the congenital anatomical status of ES. A short PP distance may suggest a small ES and poor ES function in patients with MD [5]. Previous postmortem histological studies and imaging examinations had revealed that the VA and ES were significantly smaller in patients with MD than in healthy subjects [3, 6, 21], and hypothesized that anatomical variation is one of the predisposing factors for patients with unilateral MD [5, 6]. To our knowledge, our study, for the first time, found the difference of MRI-visualized ES size between these two subtypes of hydropic ear diseases, which indicated an anatomic predisposition of ES hypoplasia or dysfunction to unilateral MD rather than to ipsilateral DEH.

ES is essential in maintaining endolymph homeostasis. The hypoplasia of ES has been assumed to compromise the absorption of endolymph, which could induce ELH in MD. Two endotypes of ES pathologies have been identified in MD, i.e., degeneration and developmental hypoplasia, which correspond to different clinical phenotypes [22, 23]. A recent radiological study has shown a preliminary correlation between bilateral ES hypoplasia and bilateral disease progression in phenotypically unilateral MD patients [24]. Other aspects, including the migraine, familial history, and comorbid autoimmune disorder, have also been suggested to contribute to the classification of the phenotypes of MD [25, 26]. In addition, the autoimmune response may also play a role in the development of MD and there is much evidence that the ES is involved in the labyrinth immuno-mediated reaction [27, 28]. It would be of great interest for future studies to examine the potential relation between the anatomical variation of ES and autoimmune response in MD patients. Although the pathogenetic role of ES in hydropic ear diseases is not fully understood, our results indicated the ES might play inconsistent roles in the pathophysiology of unilateral MD and ipsilateral DEH and multiple mechanisms are involved in the development of ELH.

In this study, differences in MRI-VA visibility were found among the affected side of unilateral MD (14.5%), ipsilateral DEH (35.7%), and control subjects (28.8%), suggesting a significant difference between at least two groups. When comparing between MD and DEH, MRI-VA visibility in MD patients seems lower than that in DEH patients, although this trend was statistically insignificant (*p* = 0.017, close to the corrected value). This may be due to the small sample size of DEH. VA is a bony canal which encloses ED and the intraosseous portion of ES. Morphological analysis of VA by radiological studies using 2D computed tomography (CT), 3D cone beam CT, and MRI has suggested a correlation between the lack of a visible ED and MD [29–31]. Besides congenital hypoplasia of VA, the calcium ion augmentation in hydropic ears may also lead to calcification and narrowing of VA. Consequently, endolymph transport may be disturbed, resulting in inner ear dysfunction [32]. Thus, our results indicated that alterations and dysfunction of the VA tend to be less severe in patients with DEH than those with MD, but this trend needs further verification by studies with larger sample size.

### No difference in MRI-visualized measurements between DEH and control

We also demonstrated no significant differences in MRI-visualized measurements between the DEH patients and control subjects. To date, the etiology and pathogenesis of DEH remains elusive [12]. Multiple factors had been proposed as responsible for the pathogenesis of DEH, including viral infections [14], intrinsic endocrine factors [33], noise exposure [34], autoimmunity [35], and head trauma [36]. Fukushima et al.suggested that profound SNHL alone did not necessarily lead to ELH in the prior deaf ears and other etiologic co-factors may disturb the homeostasis of inner ear and result in ELH [33]. Schuknecht hypothesized that an initial labyrinthine insult leads to total disruption of cochlear but relative preservation of vestibular function, and secondary atrophy or fibrous obliteration of the endolymphatic resorptive system develops after a variable latency period [37]. However, due to the low incidence of DEH, until now, direct histopathological evidence from the temporal bone examination of DEH patients was scanty [12, 14]. Based on the abovementioned hypothesis and our findings that no significant difference in the MRI-PP distance existed between DEH patients and control subjects, it is reasonable to suppose that the congenital hypoplasia of ES may not be involved in the pathogenesis of ipsilateral DEH. Other mechanisms may play a greater role, such as the vasopressin-receptor feedback system or auto-immune response in ES [35, 38, 39]. Disease duration may also impact the histopathological changes in the endolymphatic system, but the correlation between the development of hydropic symptoms and the progression of ES atrophy or obstruction is still unclear.

Taken these findings together, our study has provided preliminary radiological evidence for elucidating the underlying mechanism of MD and DEH, and in a broader sense, hydropic ear disease.

### No interaural difference of MRI-visualized measurements in DEH and MD

Based on the recent histopathological and radiological studies [40–43], we prefer the term “MRI-VA visibility” rather than “MRI-ED visibility” to reflect the high signal in the VA region in MRI, the latter one has been used in previous studies.

In this study, for the patients with ipsilateral DEH and unilateral MD, respectively, no significant differences of MRI-PP distance and MRI-VA visibility were found between affected and non-affected side. As for MRI-PP distance, Albers et al.[6] and Mateijsen et al.[5] observed no significant difference between the affected versus the non-affected ears in unilateral MD patients, which were in agreement with our findings. However, as for MRI-VA visibility, previous studies have yielded inconsistent results. In 1992, Tanioka et al.[44] first revealed the difference of MRI-visualized ED between asymptomatic and symptomatic sides in MD patients with three-dimensional Fourier transform technique with a fast low-angle shot (3DFT: FLASH). Using a similar imaging and evaluating method, Welling et al.[45] demonstrated no difference of MRI-ED visibility between the symptomatic and asymptomatic ears of patients with MD. Recently, Attyé et al.[43] further found that the MRI-VA visibility was symmetrical without significant interaural difference in patients with unilateral MD by three-dimensional fluid-attenuated inversion recovery (3D-FLAIR) with contrast media. Our results supported the theory that the hypoplasia or dysfunction of the endolymphatic resorptive system can be regarded as a predisposing factor in the pathogenesis of MD. Additionally, other mechanisms may also be involved in ELH, such as periodic overproduction of endolymph by the stria vascularis. A bi-phasic model has been proposed in MD [46], which combines two processes underlying the MD symptoms: compromised absorption of endolymph due to hypoplasia of the ES and ED and the periodic over-production of endolymph by the stria vascularis. Our results also support the hypothesis that the size of the ES is not the only operational factor in the pathogenesis of MD.

Clinically, unilateral and bilateral MD differed in many aspects such as age of onset, personal history of migraine, and family history [47]. Furthermore, previous radiological study found a significant difference of MRI-PP distance between unilateral and bilateral MD [5]. Most recently, it is reported that unilateral MD patients with bilateral hypoplastic ES, measured by high-resolution CT, have a higher risk of bilateral progression [24]. Therefore, further study with larger sample size including both uni- and bilateral MD patients was warranted to further investigate the role of anatomical variations in different subtypes of hydropic ear disease.

### No correlation between MRI-visualized measurements and clinical features or audio-vestibular findings in DEH and MD

The current study found that, for patients with unilateral MD, MRI-PP distance and MRI-VA visibility were not related to the course duration, stage of MD, SP/AP ratio, CP value, or glycerol results. Moreover, for patients with ipsilateral DEH, these radiological anatomical variations were not related to the course duration or CP value either. Similarly, Mateijsen et al.also found no relationship between MRI-PP distance and duration of disease in the MD patients [5]. In patients with MD, the results of EcochG and glycerol test can be used as traditional audiological indicators for ELH, and the caloric response is correlated with the severity of vestibular ELH (as demonstrated by gadolinium-enhanced MRI of the inner ear) [48], suggesting that caloric response may serve as a vestibular indicator for ELH [49]. These audio-vestibular variables may reflect the severity of MD indirectly and were not correlated with the radiological anatomical variations. On these grounds, it is reasonable to suppose that these MRI-visualized anatomical variations probably do not correlate with the severity and pathophysiologic status of unilateral MD and ipsilateral DEH.

### Application of MRI in hydropic ear disease

Clinically, the primary purpose of imaging examination is to exclude the retro-cochlear pathology, such as vestibular schwannoma. During the past two decades, high-resolution MRI with gadolinium as the contrast agent has been shown to provide direct evidence of ELH in the inner ear in vivo, which facilitates diagnosis of hydropic ear disease (MD, DEH, etc.) [50–52]. Meanwhile, non-enhanced MRI can also differentiate the endo- and perilymph space and is of great clinical value in detecting ELH in MD patients [53], although this statement was under debate [54]. For the patients with hydropic ear disease, the non-enhanced MRI can still provide useful radiological information to support the clinical diagnosis, rule out retro-cochlear pathology [55], and investigate the pathophysiological mechanisms [56].

Previous MRI studies of inner ear in MD patients have utilized T2-weighted images, including 3D-SPACE and 3D-FLAIR. Lorenzi et al.and Patel et al. have reported visualization of VA or ED on T2-weighted high-resolution imaging without contrast [55, 57]. Attyé et al.found no significant difference for VA characterization in the 3D-FLAIR acquisitions before and after contrast injection in MD patients [43]. Although Naganawa et al.demonstrated heavily T2-weighted 3D-FLAIR (hT2w-3D-FLAIR) more sensitive to signal alterations in the inner ear compared with 3D-FLAIR [58], further investigations in large sample cohort were needed to reach a conclusion.

This study had some limitations. Firstly, gadolinium-enhanced MRI of the inner ear, which allows for more direct visualization of ELH, was not routinely performed in our diagnostic workup for patients with DEH and MD. Secondly, our findings have demonstrated no correlation between MRI-visualized anatomical variations and the audio-vestibular ELH indicators (SP/AP ratio, CP value, and audiometric glycerol results). Interestingly, another anatomical variation of the inner ear, the VA visibility based on temporal bone CT evaluation, has been considered a predictor for the presence of ELH visualized by gadolinium-enhanced inner ear MRI in MD patients [7]. Therefore, the relationships between the anatomical variations and the presence or severity of ELH in hydropic ear disease need more investigations. Thirdly, because of the small sample size, other subtypes of hydropic ear disease, such as bilateral MD and contralateral DEH, were not enrolled in the current study. To explore the role of anatomical variations in the pathophysiology of hydropic ear diseases, further prospective controlled studies using gadolinium-enhanced MRI with other radiological indices and larger sample size are warranted.

## Conclusions

Discrepancies in MRI-visualized measurement between ipsilateral DEH and unilateral MD indicate that the anatomical variations of endolymphatic sac may be a predisposing factor in the pathogenesis in unilateral MD rather than in ipsilateral DEH.

## Supplementary Information

Below is the link to the electronic supplementary material.

Supplementary file1 (DOCX 43 KB)

## Abbreviations

3D-FLAIR: Three-dimensional fluid attenuated inversion recovery
3D-SPACE: Three-dimensional sampling perfection with application optimized contrasts using different flip angle evolutions
AAO-HNS: American Academy of Otolaryngology-Head and Neck Surgery
AP: Action potential
CP: Canal paresis
CT: Computed tomography
DEH: Delayed endolymphatic hydrops
EcochG: Electrocochleogram
ED: Endolymphatic duct
ELH: Endolymphatic hydrops
ES: Endolymphatic sac
FOV: Field of view
ICC: Interclass correlation efficient
IQR: Interquartile range
MD: Ménière’s disease
MRI: Magnetic resonance imaging
MRI-PP distance: Distance between the vertical part of the posterior semicircular canal and the posterior fossa visualized by MRI
PACS: Picture archiving and communication system
SD: Standard deviations
SNHL: Sensorineural hearing loss
SP: Summating potential
SPV_max_: Maximum slow phase velocity
VA: Vestibular aqueduct

## Declarations

### Guarantor

The scientific guarantor of this publication is Bo Liu.

### Statistics and biometry

Pro. Meixia Lu kindly provided statistical advice for this manuscript.

### Methodology

• Retrospective

• case–control study

• performed at one institution
