# Supplementary material for: Anatomical variation of inner ear may be a predisposing factor for unilateral Ménière’s disease rather than for ipsilateral delayed endolymphatic hydrops
Source: Eur Radiol. 2022 Jan 3;32(5):3553–64. doi: 10.1007/s00330-021-08430-7 (PMC9038801; doi:10.1007/s00330-021-08430-7)
Supplement: Supplementary file 1 — Supplementary file1 (DOCX 43 KB) [file 330_2021_8430_MOESM1_ESM.docx]

| **Supplementary Table 1**: MR scanning protocol | | | |
| --- | --- | --- | --- |
| Scanning Parameters（3.0T） | T1 weighted imaging | T2 weighted imaging | 3D-SPACE |
| Plane | sagittal and axial | axial | axial |
| TR (ms) | 300 | 9000 | 1000 |
| TE (ms) | 2 | 90 | 135 |
| Fat saturation | / | TIR | / |
| slice thickness(mm) | 5 | 5 | 0.5 |
| Slices(no.) | 15 | 17 | 56 |
| FOV (mm^2^) | 250×250 | 220×200 | 200×200 |
| matrix | 320×320 | 640×640 | 384×384 |
| Averages | 1 | 1 | 2 |
| Bandwidth (Hz/Px) | 330 | 287 | 289 |
| 3D-SPACE: Three-dimensional sampling perfection with application optimized contrasts using different flip angle evolutions; TR: Repetition Time; TE: Echo Time; FOV: Field of View. | | | |
|  |  |  |  |
|  |  |  |  |

| **Supplementary Table 2:** The data of MRI-PP distance and VA visualization in DEH, MD and control groups | | | | | | | | | |
| --- | --- | --- | --- | --- | --- | --- | --- | --- | --- |
|  |  | 1st radiologist（L.P） | | | | 2nd radiologist (L.J) | | | |
|  |  | MRI-PP distance/mm | | MRI-VA* | | MRI-PP distance/mm | | MRI-VA* | |
|  |  | left | right | left | right | left | right | left | right |
| DEH | 1 | 6.29 | 5.05 | 1 | 0 | 4.8 | 5.72 | 1 | 1 |
|  | 2 | 0.66 | 0.67 | 0 | 0 | 0.46 | 0.7 | 0 | 0 |
|  | 3 | 1.95 | 1.71 | 0 | 0 | 1.89 | 1.82 | 0 | 0 |
|  | 4 | 2.64 | 1.16 | 0 | 0 | 2.84 | 1.26 | 0 | 0 |
|  | 5 | 2.4 | 3.06 | 1 | 1 | 2.83 | 3.14 | 1 | 1 |
|  | 6 | 0.69 | 1.46 | 0 | 0 | 0.94 | 1.57 | 0 | 0 |
|  | 7 | 4.15 | 4 | 1 | 0 | 4.38 | 4.23 | 1 | 0 |
|  | 8 | 1 | 0.51 | 1 | 0 | 0.77 | 0.61 | 1 | 0 |
|  | 9 | 3.68 | 4.04 | 1 | 1 | 3.49 | 4.36 | 1 | 1 |
|  | 10 | 4.61 | 4.68 | 0 | 0 | 4.85 | 5.18 | 0 | 0 |
|  | 11 | 5.02 | 5.24 | 0 | 1 | 4.97 | 5.1 | 0 | 0 |
|  | 12 | 3.42 | 4.12 | 0 | 0 | 3.16 | 4.1 | 0 | 0 |
|  | 13 | 0.76 | 0.17 | 0 | 0 | 0.77 | 0.18 | 0 | 0 |
|  | 14 | 4.82 | 2.56 | 0 | 1 | 4.57 | 2.8 | 0 | 0 |
|  | 15 | 2.6 | 2.49 | 0 | 0 | 2.34 | 2.24 | 0 | 0 |
|  | 16 | 2.01 | 4 | 0 | 1 | 1.84 | 4.05 | 0 | 1 |
|  | 17 | 1.99 | 1.38 | 1 | 1 | 2.05 | 1.45 | 1 | 1 |
|  | 18 | 4.77 | 4.07 | 1 | 1 | 4.85 | 3.87 | 1 | 1 |
|  | 19 | 1.36 | 1.04 | 0 | 0 | 1.63 | 1.11 | 0 | 0 |
|  | 20 | 2.94 | 2.62 | 1 | 1 | 2.97 | 2.53 | 1 | 1 |
|  | 21 | 3.27 | 3.37 | 0 | 0 | 3.33 | 3.43 | 0 | 0 |
|  | 22 | 0.84 | 0.72 | 0 | 0 | 0.9 | 0.51 | 0 | 0 |
|  | 23 | 1.57 | 1.1 | 0 | 0 | 1.35 | 1.04 | 0 | 0 |
|  | 24 | 5.04 | 3.62 | 0 | 1 | 4.41 | 3.49 | 0 | 1 |
|  | 25 | 2.64 | 1.79 | 1 | 1 | 2.54 | 1.86 | 1 | 1 |
|  | 26 | 1.49 | 1.35 | 0 | 0 | 1.7 | 1.19 | 0 | 0 |
|  | 27 | 4.21 | 3.99 | 0 | 0 | 4.18 | 3.81 | 0 | 0 |
|  | 28 | 6.25 | 3.94 | 0 | 0 | 6.63 | 3.46 | 0 | 0 |
| MD | 1 | 2.76 | 2.26 | 0 | 0 | 2.5 | 2.11 | 0 | 0 |
|  | 2 | 1.32 | 2.04 | 0 | 0 | 1.15 | 2.1 | 0 | 0 |
|  | 3 | 1.41 | 1.62 | 0 | 0 | 1.2 | 1.36 | 0 | 0 |
|  | 4 | 0.1 | 0.4 | 0 | 0 | 0.3 | 0.3 | 0 | 0 |
|  | 5 | 4.01 | 5.7 | 0 | 0 | 4.26 | 5.89 | 0 | 0 |
|  | 6 | 2.56 | 2.76 | 0 | 0 | 2.62 | 3.08 | 0 | 0 |
|  | 7 | 1.2 | 1.3 | 0 | 1 | 0.98 | 1.23 | 0 | 1 |
|  | 8 | 1.95 | 1.85 | 1 | 1 | 1.88 | 1.68 | 1 | 0 |
|  | 9 | 3.66 | 3.43 | 0 | 1 | 3.65 | 3.48 | 1 | 1 |
|  | 10 | 2.37 | 2.31 | 0 | 0 | 2.36 | 2.53 | 0 | 0 |
|  | 11 | 3.65 | 1.28 | 0 | 0 | 3.71 | 1.21 | 0 | 0 |
|  | 12 | 1.51 | 1.54 | 0 | 0 | 1.36 | 1.55 | 0 | 0 |
|  | 13 | 0.71 | 0.33 | 0 | 0 | 0.78 | 0.35 | 0 | 0 |
|  | 14 | 0.82 | 1.4 | 0 | 0 | 0.93 | 1.8 | 0 | 0 |
|  | 15 | 1.61 | 2.89 | 0 | 1 | 1.67 | 2.77 | 0 | 0 |
|  | 16 | 3.04 | 1.2 | 1 | 1 | 2.59 | 1.23 | 0 | 1 |
|  | 17 | 1.24 | 1.15 | 0 | 0 | 1.09 | 1.16 | 0 | 1 |
|  | 18 | 3.6 | 3.24 | 0 | 0 | 3.1 | 3.31 | 0 | 0 |
|  | 19 | 0.78 | 1.78 | 0 | 0 | 0.6 | 1.54 | 1 | 1 |
|  | 20 | 1.79 | 4.18 | 0 | 0 | 1.54 | 4.14 | 0 | 0 |
|  | 21 | 1.71 | 1.09 | 0 | 0 | 1.84 | 1.01 | 0 | 0 |
|  | 22 | 4.66 | 4.77 | 0 | 0 | 4.48 | 4.64 | 0 | 0 |
|  | 23 | 0.62 | 0.52 | 0 | 0 | 0.5 | 0.7 | 0 | 0 |
|  | 24 | 0.59 | 0.47 | 0 | 0 | 0.68 | 0.88 | 0 | 0 |
|  | 25 | 0.22 | 0.1 | 1 | 0 | 0.4 | 0.1 | 0 | 0 |
|  | 26 | 0.45 | 0.32 | 0 | 0 | 0.4 | 0.3 | 0 | 0 |
|  | 27 | 0.8 | 1.05 | 0 | 0 | 0.81 | 1.13 | 0 | 0 |
|  | 28 | 0.57 | 0.5 | 0 | 0 | 0.79 | 0.59 | 0 | 0 |
|  | 29 | 1.47 | 2.17 | 0 | 0 | 1.51 | 2.19 | 0 | 0 |
|  | 30 | 0.92 | 3.16 | 0 | 0 | 1.19 | 3.08 | 0 | 0 |
|  | 31 | 1.68 | 0 | 1 | 1 | 1.66 | 0 | 1 | 1 |
|  | 32 | 0.47 | 0.77 | 0 | 0 | 0.55 | 0.57 | 0 | 0 |
|  | 33 | 0.88 | 0.69 | 1 | 0 | 0.7 | 0.75 | 0 | 0 |
|  | 34 | 3 | 1.78 | 1 | 0 | 2.43 | 1.9 | 1 | 0 |
|  | 35 | 2.36 | 2.7 | 0 | 0 | 2.17 | 2.41 | 0 | 0 |
|  | 36 | 0.35 | 0.48 | 0 | 1 | 0.33 | 0.64 | 0 | 0 |
|  | 37 | 0.75 | 0.61 | 0 | 0 | 0.52 | 0.64 | 0 | 0 |
|  | 38 | 1.29 | 2.6 | 1 | 1 | 1.03 | 2.73 | 1 | 1 |
|  | 39 | 3.67 | 3.26 | 0 | 1 | 3.73 | 3.15 | 0 | 1 |
|  | 40 | 2.15 | 3.11 | 0 | 1 | 2.04 | 3.35 | 0 | 0 |
|  | 41 | 0.69 | 0.93 | 1 | 1 | 0.84 | 0.89 | 1 | 1 |
|  | 42 | 2.51 | 4.3 | 0 | 0 | 2.48 | 4.15 | 0 | 0 |
|  | 43 | 1.59 | 1.5 | 1 | 0 | 1.5 | 1.43 | 1 | 0 |
|  | 44 | 1.54 | 1.81 | 0 | 0 | 1.55 | 1.85 | 0 | 0 |
|  | 45 | 0.5 | 1.21 | 0 | 0 | 0.75 | 0.95 | 0 | 0 |
|  | 46 | 1.56 | 1.89 | 1 | 1 | 1.49 | 1.74 | 1 | 1 |
|  | 47 | 1.8 | 2.26 | 0 | 0 | 1.85 | 2.17 | 0 | 0 |
|  | 48 | 2.43 | 2.78 | 1 | 1 | 1.75 | 2.64 | 1 | 1 |
|  | 49 | 1.12 | 1.2 | 0 | 0 | 1.03 | 1.2 | 0 | 0 |
|  | 50 | 0.21 | 0.11 | 0 | 0 | 0.17 | 0.17 | 0 | 0 |
|  | 51 | 3.65 | 4.19 | 0 | 0 | 3.93 | 4.05 | 0 | 0 |
|  | 52 | 0.39 | 0.88 | 0 | 0 | 0.53 | 0.73 | 0 | 0 |
|  | 53 | 1.37 | 2.14 | 0 | 0 | 1.45 | 1.97 | 0 | 0 |
|  | 54 | 0.94 | 1.39 | 0 | 0 | 0.77 | 1.42 | 0 | 0 |
|  | 55 | 1.28 | 1.54 | 0 | 0 | 1.99 | 1.58 | 0 | 0 |
|  | 56 | 2.43 | 2.37 | 1 | 0 | 2.22 | 2.5 | 1 | 0 |
|  | 57 | 1.53 | 1.75 | 1 | 0 | 1.77 | 1.52 | 1 | 0 |
|  | 58 | 0.38 | 0.96 | 0 | 0 | 0.63 | 0.9 | 0 | 0 |
|  | 59 | 2.57 | 2.99 | 1 | 1 | 2.71 | 2.83 | 1 | 1 |
|  | 60 | 2.86 | 2.09 | 0 | 0 | 2.68 | 2.01 | 0 | 0 |
|  | 61 | 2.85 | 3.98 | 0 | 1 | 4.14 | 3.8 | 1 | 1 |
|  | 62 | 2.45 | 2.19 | 0 | 0 | 2.31 | 1.91 | 0 | 0 |
|  | 63 | 3.49 | 4.5 | 0 | 0 | 3.15 | 4.68 | 0 | 0 |
|  | 64 | 0.39 | 1.33 | 0 | 0 | 0.64 | 1.03 | 0 | 0 |
|  | 65 | 0.91 | 2.9 | 0 | 0 | 1.1 | 3.04 | 0 | 0 |
|  | 66 | 4.35 | 4.15 | 0 | 0 | 4.02 | 4.28 | 0 | 0 |
|  | 67 | 6.17 | 4.49 | 0 | 0 | 7.37 | 4.3 | 0 | 0 |
|  | 68 | 2.04 | 1.57 | 0 | 0 | 2.04 | 1.75 | 0 | 0 |
|  | 69 | 1.17 | 2.95 | 0 | 0 | 1.39 | 2.83 | 0 | 0 |
|  | 70 | 1.48 | 2.07 | 0 | 0 | 1.63 | 1.85 | 0 | 0 |
|  | 71 | 1.35 | 1.17 | 0 | 1 | 1.71 | 1.25 | 0 | 1 |
|  | 72 | 1.66 | 2.45 | 0 | 0 | 1.69 | 2.1 | 0 | 0 |
|  | 73 | 1.82 | 2.4 | 0 | 0 | 1.91 | 2.29 | 0 | 0 |
|  | 74 | 1.37 | 1.44 | 0 | 0 | 1.43 | 1.58 | 0 | 0 |
|  | 75 | 1.2 | 3.15 | 0 | 0 | 1.49 | 3.04 | 0 | 0 |
|  | 76 | 0.64 | 0.56 | 0 | 0 | 0.91 | 0.72 | 0 | 0 |
| control | 1 | 3.13 | 4.04 | 0 | 0 | 2.91 | 3.54 | 0 | 0 |
|  | 2 | 2.84 | 2.13 | 0 | 0 | 2.91 | 1.88 | 0 | 0 |
|  | 3 | 3.78 | 3.44 | 0 | 1 | 3.2 | 3.36 | 1 | 1 |
|  | 4 | 4.66 | 2.7 | 0 | 0 | 4.56 | 3.05 | 0 | 0 |
|  | 5 | 1.37 | 0.84 | 1 | 1 | 1.37 | 0.69 | 1 | 1 |
|  | 6 | 1.98 | 1.4 | 0 | 0 | 1.87 | 1.67 | 0 | 0 |
|  | 7 | 1 | 2.04 | 1 | 0 | 0.93 | 2.1 | 1 | 0 |
|  | 8 | 2.29 | 2.12 | 1 | 1 | 2.05 | 1.92 | 1 | 1 |
|  | 9 | 4.58 | 3.25 | 0 | 0 | 4.14 | 3.33 | 0 | 1 |
|  | 10 | 2.57 | 2.57 | 1 | 1 | 2.27 | 2.49 | 1 | 1 |
|  | 11 | 0.9 | 0.87 | 1 | 0 | 0.83 | 0.75 | 1 | 0 |
|  | 12 | 1.83 | 1.23 | 0 | 0 | 1.8 | 1.34 | 0 | 0 |
|  | 13 | 1.54 | 1.38 | 0 | 0 | 1.6 | 1.32 | 0 | 0 |
|  | 14 | 1.47 | 1.6 | 0 | 0 | 1.58 | 1.77 | 0 | 0 |
|  | 15 | 0 | 0.4 | 0 | 1 | 0 | 0.35 | 0 | 0 |
|  | 16 | 3.27 | 3.53 | 0 | 0 | 3.23 | 3.36 | 0 | 0 |
|  | 17 | 1.84 | 2.07 | 0 | 0 | 2.04 | 2.58 | 0 | 0 |
|  | 18 | 0.96 | 1.58 | 0 | 0 | 1.03 | 1.46 | 0 | 0 |
|  | 19 | 3.49 | 4.92 | 1 | 0 | 4.53 | 3.32 | 0 | 0 |
|  | 20 | 1.54 | 0.89 | 0 | 0 | 1.5 | 0.96 | 0 | 0 |
|  | 21 | 4.17 | 1.94 | 0 | 0 | 4.25 | 1.82 | 0 | 0 |
|  | 22 | 3.46 | 4.9 | 0 | 0 | 3.4 | 4.4 | 0 | 0 |
|  | 23 | 5.16 | 3.1 | 0 | 1 | 5.01 | 3.06 | 0 | 1 |
|  | 24 | 1.23 | 1.87 | 0 | 0 | 0.98 | 1.82 | 0 | 0 |
|  | 25 | 2.67 | 1.95 | 0 | 0 | 2.32 | 1.79 | 0 | 0 |
|  | 26 | 3.09 | 3.06 | 1 | 1 | 2.7 | 3.13 | 1 | 0 |
|  | 27 | 1.06 | 1.95 | 0 | 0 | 1.24 | 1.86 | 0 | 0 |
|  | 28 | 2.15 | 2.7 | 0 | 0 | 2.26 | 2.45 | 0 | 0 |
|  | 29 | 1.93 | 2.29 | 0 | 1 | 1.88 | 2.25 | 0 | 1 |
|  | 30 | 2.96 | 3.2 | 0 | 0 | 2.74 | 3.06 | 0 | 0 |
|  | 31 | 1.16 | 0.97 | 0 | 0 | 1.19 | 1.07 | 0 | 0 |
|  | 32 | 3.31 | 1.4 | 1 | 1 | 3.01 | 1.49 | 1 | 1 |
|  | 33 | 1.03 | 3.45 | 1 | 1 | 1.16 | 3.74 | 1 | 1 |
|  | 34 | 3.8 | 3.92 | 0 | 0 | 3.46 | 3.6 | 0 | 0 |
|  | 35 | 0.75 | 1.47 | 0 | 0 | 0.56 | 1.42 | 0 | 0 |
|  | 36 | 2.15 | 2.04 | 0 | 0 | 2.03 | 2.15 | 0 | 0 |
|  | 37 | 0.66 | 1.31 | 1 | 1 | 0.77 | 1.26 | 1 | 1 |
|  | 38 | 3.82 | 3.38 | 0 | 0 | 3.67 | 3.3 | 0 | 0 |
|  | 39 | 1.05 | 2.39 | 1 | 1 | 1.2 | 2.31 | 1 | 1 |
|  | 40 | 1.22 | 2.37 | 0 | 0 | 1.25 | 2.15 | 0 | 0 |
|  | 41 | 2.58 | 2.69 | 1 | 1 | 2.82 | 2.72 | 1 | 1 |
|  | 42 | 4.21 | 4.28 | 0 | 1 | 4.16 | 4.03 | 0 | 1 |
|  | 43 | 1.98 | 2.29 | 0 | 0 | 2.3 | 2.78 | 0 | 0 |
|  | 44 | 1.7 | 3.25 | 0 | 0 | 2 | 3.48 | 0 | 0 |
|  | 45 | 2.46 | 1.62 | 0 | 0 | 2.39 | 1.67 | 0 | 0 |
|  | 46 | 3.91 | 4.12 | 1 | 1 | 3.84 | 3.94 | 1 | 1 |
|  | 47 | 1.25 | 2.36 | 0 | 0 | 1.49 | 2.29 | 0 | 0 |
|  | 48 | 2.94 | 1.88 | 0 | 0 | 2.76 | 1.96 | 0 | 0 |
|  | 49 | 3.72 | 2.34 | 1 | 0 | 4.01 | 2.3 | 1 | 0 |
|  | 50 | 2.52 | 2.71 | 0 | 0 | 2.1 | 2.67 | 0 | 0 |
|  | 51 | 1.23 | 1.88 | 1 | 0 | 1.15 | 1.99 | 1 | 0 |
|  | 52 | 1.27 | 1.52 | 1 | 1 | 1.19 | 1.41 | 1 | 1 |
|  | 53 | 6.06 | 7.11 | 0 | 0 | 5.94 | 6.88 | 0 | 0 |
|  | 54 | 2.1 | 1.83 | 1 | 0 | 2.26 | 2 | 1 | 0 |
|  | 55 | 1.12 | 1.36 | 0 | 0 | 1.32 | 1.24 | 0 | 0 |
|  | 56 | 4.07 | 3.3 | 0 | 1 | 3.89 | 3.19 | 0 | 1 |
|  | 57 | 3.74 | 4.49 | 0 | 0 | 3.92 | 4.53 | 0 | 0 |
|  | 58 | 1.41 | 1.85 | 1 | 1 | 1.79 | 1.9 | 1 | 1 |
|  | 59 | 2.84 | 1.6 | 0 | 1 | 2.58 | 1.67 | 0 | 0 |
| MD: Ménière's disease; DEH: delayed endolymphatic hydrops; MRI-PP Distance: distance between the vertical part of the posterior semicircular canal and the posterior fossa visualized by MRI; VA: vestibular aqueduct.  *:"1"refers to visualization of the vestibular aqueduct; "0"refers to non-visualization of the vestibular aqueduct. | | | | | | | | | |
